# Idiopathic Multicentric Castleman Disease Occurring Shortly after mRNA SARS-CoV-2 Vaccine

**DOI:** 10.3390/vaccines10101725

**Published:** 2022-10-15

**Authors:** Christian Hoffmann, Thomas Wechselberger, Heinz Drexel, Susanne Dertinger, Stefan Dirnhofer, Sheila K. Pierson, David C. Fajgenbaum, Andreas Kessler

**Affiliations:** 1 ICH Study Center Hamburg, 20095 Hamburg, Germany; 2 Campus Kiel, University of Schleswig Holstein, 24105 Kiel, Germany; 3 Department of Hematology, Oncology, Palliative Medicine, Hemostaseology, Provincial Hospital Bregenz, 6900 Bregenz, Austria; 4 Vorarlberg Institute for Vascular Investigation and Treatment (*VIVIT*), 68540 Feldkirch, Austria; 5 Department of Pathology, Academic Teaching Hospital Feldkirch, 6800 Feldkirch, Austria; 6 Department of Pathology, University Hospital Basel, Schoenbeinstr. 40, CH-4031 Basel, Switzerland; 7 Department of Medicine, Division of Translational Medicine and Human Genetics, Perelman School of Medicine, University of Pennsylvania, Philadelphia, PA 19019, USA

**Keywords:** Idiopathic Multicentric Castleman Disease, mRNA SARS-CoV-2 vaccine, TAFRO syndrome, siltuximab

## Abstract

Idiopathic Multicentric Castleman Disease (iMCD) is a potentially life-threatening systemic disease whose complex symptomatology is due to cytokine dysregulation. We, herein, present a case of severe iMCD occurring in a previously healthy young man shortly after mRNA SARS-CoV-2 vaccination, responding to interleukin-6 blockade with siltuximab. Six months after the completion of siltuximab, the patient remained without any signs of iMCD or inflammation, indicating a temporal trigger of the disease. This case not only adds to the potential pathogenetic spectrum of MCD, but also extends the clinical picture of potential but rare adverse events following COVID-19 immunization.

## 1. Introduction

With the global campaigns for vaccinations against severe acute respiratory syndrome-coronavirus disease 2 (SARS-CoV-2), rare and novel vaccine-associated, systemic inflammatory and immune-mediated adverse events are increasingly being reported [1]. Idiopathic Multicentric Castleman Disease (iMCD) is a potentially life-threatening systemic disease whose complex symptomatology is due to cytokine dysregulation. The overproduction of interleukin-6 (IL-6) is a known driver in some patients. Though etiology is unknown, proposed mechanisms include a pathogen/viral hypothesis, paraneoplastic hypothesis and autoimmune hypothesis [2]. Severity ranges from few clinical and laboratory abnormalities to multi-organ failure. Clinically distinct variants have been described for iMCD, the most severe of which is TAFRO syndrome (thrombocytopenia, anasarca, fever, reticulin fibrosis, renal failure and organomegaly) [3]. Siltuximab—an anti-IL-6, chimeric monoclonal antibody—is the only treatment approved for iMCD in the European Union and the United States and is recommended as the first-line treatment based on the phase II trial, which demonstrated a 34% tumor and symptomatic response [4,5].

## 2. Case Report

A 20-year-old, previously healthy young man presented at the emergency unit 18 days after receiving a second dose of the mRNA vaccine BNT162b2. Approximately two weeks prior to admission, he had noticed swollen axillary lymph nodes, fever of up to 39.5 degrees centigrade, loss of appetite, malaise, weakness and exertional dyspnea. He reported no previous adverse reactions to vaccines nor allergies to food or drugs.

His medical history included a congenital heart defect with a double-outlet right ventricle with subvalvular pulmonary and aortic stenosis that had undergone successful cardiac surgery at the age of 11 months. He had not experienced any COVID-19 symptoms in the previous months nor received a positive test in the past for SARS-CoV-2. 

On admission, physical examination was notable for anasarca, petechia and palpable lymphadenopathy. Temperature was 37.2 degrees and inflammatory parameters such as C-reactive protein (CRP 135, normal range < 5 mg/L) and procalcitonin (2.6, normal range < 0.1) were markedly elevated. IL-6 reached a level of 45.5 pg/mL (normal range < 7.0 pg/mL). Laboratory test results were also notable for thrombocytopenia (16,000 per µL, repeated on a sample from citrate-anticoagulated blood), leukopenia (2.400 per µL), anemia (9.4 mg/dL), hypoalbuminemia (2.6 g/dL, normal range 3.5–4.5 g/dL) and a very moderate impairment of the renal function (creatinine 1.25 mg/dL). Other laboratory values, including immune globulins as well as parameters of the liver function were in normal ranges. Further tests revealed no evidence for any autoimmune disease or viral infections, including HIV, CMV, EBV, hepatitis A, B, C, hantavirus, HHV-8 and SARS-CoV-2 (PCR and serologic tests for nucleocapsid and spike IgG). Anti-platelet factor 4 antibodies were also negative.

**Figure 1:**
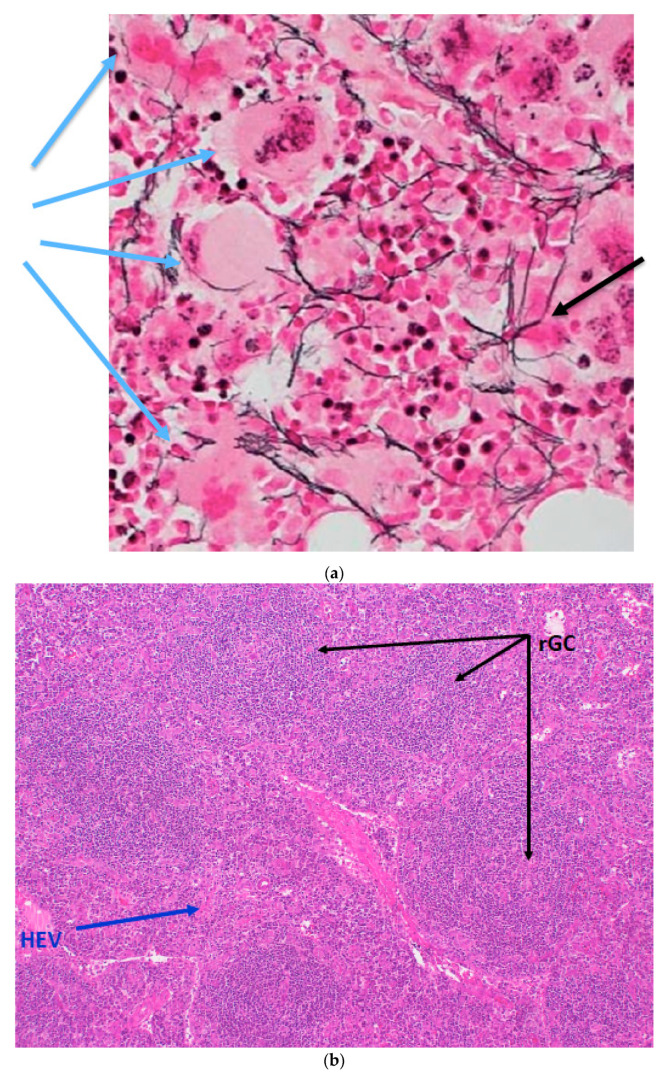

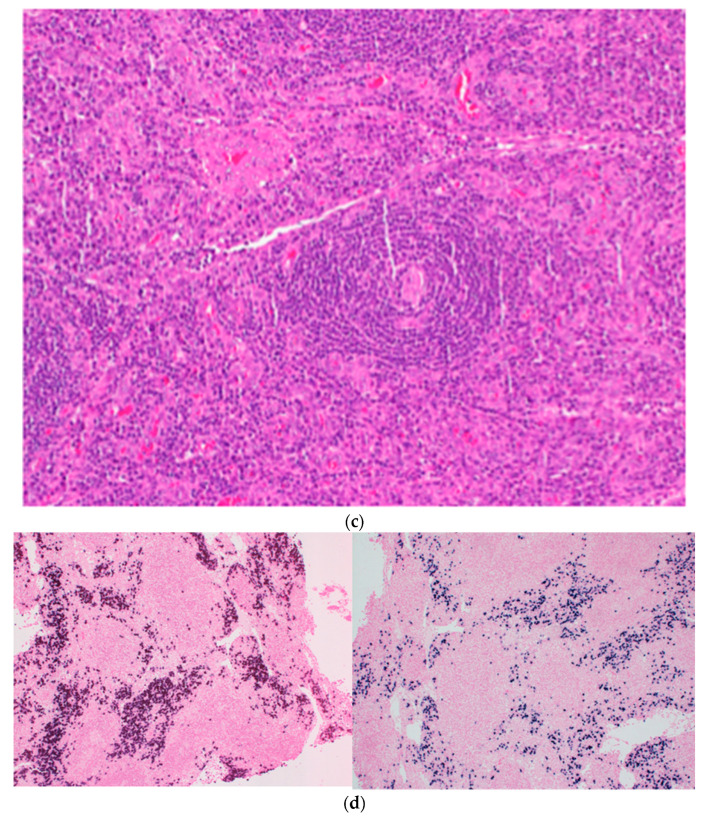
(**a**) Reticulin stain of bone marrow showing focally increased megakaryopoiesis (e.g., blue arrows) with little fibrosis (e.g., black arrow) (×40). (**b**) Regressed germinal centers (rGCs), interfollicular high endothelial vessels (HEV) and plasmocytosis (hematoxylin and eosin staining × 200). (**c**) “Onion skinning” created by expanded mantle zones with concentrically arranged lymphocytes around the regressed GC (hematoxylin and eosin staining × 100). (**d**) Immunohistochemistry (ICH, × 100) staining for kappa (**right**) and lambda light chains (**left**). Ig Kappa:Ig Lambda = 1:5.

CT and MRT imaging displayed multiple supraclavicular, axillary and abdominal lymph nodes that were enlarged up to 34 × 15 mm, as well as ascites, pleural effusions and hepatosplenomegaly. A transthoracic echocardiogram revealed a normal ventricular function and no evidence for endocarditis. Bone marrow showed a hypercellular picture, with a left-shifted macrocytic erythropoiesis, increased megakaryopoesis as well as some accentuation of reticulin fibers that were considered grade 1, according to the WHO 2017 criteria for the grading of reticulin fibrosis [6] (Figure 1a). In situ hybridization for Ig kappa and Ig lambda yielded no evidence of monoclonal plasma cell proliferation.

Since empirical treatment with antibiotics (first-line amoxicillin and clavulanic acid, later changed to piperacillin and tazobactam) and dexamethasone (for a presumed idiopathic thrombocytopenic purpura) did not induce a clinical effect, the decision for a lymph node extirpation was made in order to establish a definitive diagnosis.

The histology of a right axillary lymph node revealed polyclonal plasma cell proliferation, atrophic germinal centers and proliferation of high-endothelial venules, which was in line with the diagnosis criteria of iMCD [7] (Figure 1b–d). There was no evidence for IgG4-related disease or HHV-8.

**Figure 2:**
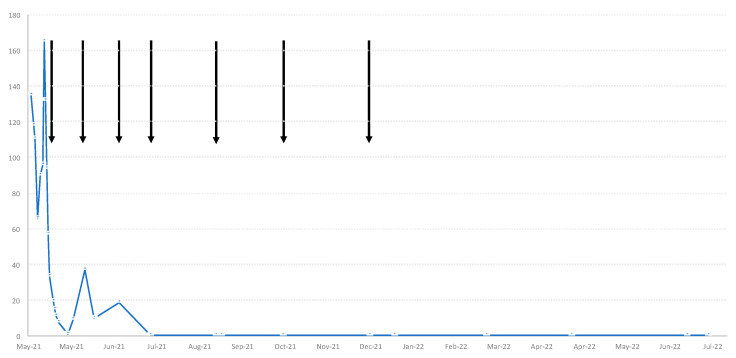
C-reactive protein (CRP, mg/L over time and siltuximab infusions (arrows).

Facing the diagnosis of a severe iMCD, the decision was made to treat the patient with intravenous siltuximab which was dosed at 11 mg/kg, as approved, every three weeks. Intervals between cycles were later extended to six weeks. Siltuximab treatment led to a rapid improvement in his clinical condition, remission of fluid accumulation and normalization of inflammation markers and other parameters. In view of the low risk for severe COVID-19 and the experience gained in this particular case, the patient was refrained from a further vaccination. CRP over time and siltuximab cycles are depicted in Figure 2. Given the absence of any iMCD signs or inflammation, siltuximab maintenance therapy was halted after a total of seven infusions. Almost twelve months after diagnosis and more than six months after completion of IL-6 blockade, the patient remained symptomless and in an excellent condition.

## 3. Discussion

Though rare systemic inflammatory and immune-mediated adverse events have been reported following SARS-CoV-2 immunizations [8,9,10,11], we report here the first case of biopsy-confirmed iMCD that appears to be associated with SARS-CoV-2 immunizations. The clinical picture of our patient fulfilled the proposed consensus criteria for iMCD [7]. These require the presence of both major criteria (characteristic lymph node histopathology and multicentric lymphadenopathy) as well as at least two minor criteria. Of the 11 proposed minor criteria, 6 were present, including elevated CRP, thrombocytopenia, hypoalbuminemia, constitutional symptoms, hepatosplenomegaly and effusions. The patient also fulfilled the proposed international definition for the TAFRO syndrome, which is considered as an aggressive subtype of iMCD, requiring four clinical criteria (thrombocytopenia, anasarca, fever/hyperinflammatory status, organomegaly), renal dysfunction or characteristic bone marrow findings [12] and a lymph node with features consistent with iMCD [8]. Of note, reticulin fibrosis in the bone marrow was mild in our case.

Although the etiology of iMCD is unknown, increased IL-6 signaling is the established driver of symptomatology and pathogenesis in a portion of patients [2]. The cytokine storm that drives iMCD is hypothesized to be caused by an uncontrolled infection (pathogen hypothesis), auto-antibodies or auto-reactive T cells associated with predisposing germline mutations (autoimmune hypothesis), germline mutations in genes regulating inflammation (autoinflammatory hypothesis) and/or somatic mutations in monoclonal lymph node cells that lead to ectopic cytokine secretion (paraneoplastic hypothesis). Preliminary findings from a patient-based survey did not indicate a markedly increased inflammatory response to SARS-CoV-2 infection and vaccination in CD patients [13]. Of the 87 responding patients who had received at least one vaccine dose, 59% reported vaccine side effects, but these had been generally mild and none required hospitalization. Treatment for CD was paused for seven of the responding patients during the vaccination period, presumably to increase the likelihood of a robust response. No difference in their vaccination response was noted [13].

To our knowledge, the case presented here is the first confirmed case of iMCD occurring in a previously healthy young man shortly after the mRNA SARS-CoV-2 vaccine. Though it remains difficult to establish causality, we propose that the likely trigger of iMCD in our case was the recent vaccination. The temporal association between vaccination and iMCD was striking and an alternative disease trigger was not identified despite a thorough investigation. As seen with other vaccines [14], COVID-19 vaccines may not only induce B- and T-cell activation, but also pronounced increases in several key inflammatory mediators, including IL-6, which was elevated in our patient. In one recent study, the increase in IL-6 was higher with mRNA vaccines, compared to adenovirus-vector-based vaccines [15]. Another recent study found a correlation between proinflammatory cytokine levels in sera and adverse reactions after mRNA vaccination, indicating that proinflammatory cytokines are a cause of adverse reactions after vaccination [15]. Of note, even six months after the completion of IL-6 blockade, our patient remained healthy without any signs of iMCD or inflammation, indicating only a temporal trigger at the onset of the disease.

A recent case study reported a TAFRO syndrome independent of iMCD-like lymph node histopathological changes that occurred in a Japanese patient one day subsequent to the second dose of the BNT162b2 mRNA COVID-19 vaccine [16]. IL-6 was likewise elevated in this 42-year-old man who was treated with the IL-6 receptor blocker, tocilizumab, as well as steroids, rituximab and antibiotics. The patient died from the disease after approximately three months. Though the authors report that the post-autopsy lymph node did not demonstrate iMCD-like changes, it is unclear if the biopsied lymph node was enlarged, and it is possible that these non-diagnostic results are due to a therapeutic intervention between TAFRO diagnosis and death (94 days). It might therefore be possible that we report the second case of iMCD-TAFRO triggered by the COVID-19 vaccine, even though it is the first biopsy-proven case.

Some other small case series have reported de novo cases of hemophagocytic lymphohistiocytosis (HLH) [9,17,18,19,20,21] and adult-onset Still disease (AOSD) [22,23], occurring shortly after the administration of different types of COVID-19 vaccines. Both entities show some clinical and pathologic overlapping with iMCD and must be excluded before making the diagnosis of iMCD. However, lymphadenopathy was rarely seen, and we are unaware of any lymph node extirpation in these cases. In addition, our patient neither showed myalgias, exanthema nor other signs indicative of HLH or AOSD. He also had no gastrointestinal symptoms, no evidence of mucocutaneous inflammation (rash, conjunctivitis, oromucosal changes) or cardiac involvement that may have indicated a multisystem inflammatory syndrome (MIS). MISs are febrile syndromes with elevated inflammatory markers that usually manifest a few weeks after a SARS-CoV-2 infection; the existence of post-vaccination MIS (MIS-V) has been speculated about. However, the current understanding of MIS is still limited, and it is unclear if MIS may occur during different post-vaccination scenarios and with various types of vaccines [24]. In our case, there was no evidence for a prior or current SARS-CoV-2 infection.

A recent review reported on high numbers of lymphadenopathy post-COVID-19 vaccination with increased FDG uptake that may be falsely attributed to oncological disorders [25]. In general, adenopathy occurs ipsilateral to the vaccination site and recovers spontaneously within days. We propose that in cases of post-vaccination multisystem inflammatory syndromes with persistent and bilateral lymphadenopathy, as seen in our case, a lymph node extirpation should be considered.

In conclusion, the presented case could, if confirmed by others, not only extend the potential pathogenetic spectrum of iMCD, but also broaden the clinical picture of potential adverse events following COVID-19 immunization. This histologically confirmed case of an mRNA vaccine-induced iMCD with TAFRO syndrome is remarkable because of its suspected etiology, the rapid response on treatment with siltuximab and the lack of recurrence of the disease for more than half a year follow-up period.

We would also like to underline that the potential vaccine complications, as described in our case, do not diminish the overwhelming positive risk–benefit ratio of licensed COVID-19 vaccines. Such rare adverse events should not encourage vaccine hesitancy or skepticism.

## Data Availability

Not applicable.
